# Characterization and phylogenetic analysis of the complete chloroplast genome sequence of *xerophyta retinervis* (velloziaceae)

**DOI:** 10.1080/23802359.2022.2067500

**Published:** 2022-04-21

**Authors:** Junyi Zhang, Min Liao, Xiong Li, Bo Xu

**Affiliations:** a CAS Key Laboratory of Mountain Ecological Restoration and Bioresource Utilization & Ecological Restoration and Biodiversity Conservation Key Laboratory of Sichuan Province, Chengdu Institute of Biology, Chinese Academy of Sciences, Chengdu, China; b College of Life Sciences, Chongqing Normal University, Chongqing, China; c College of Life Sciences, University of Chinese Academy of Sciences, Beijing, China

**Keywords:** Chloroplast genome, phylogeny, *Xerophyta retinervis*

## Abstract

*Xerophyta retinervis* has great ornamental value and numerous traditional uses. This study reported its first complete chloroplast genome sequence, which was 155,109 bp in length, including a pair of inverted repeat regions (IRs) (27,093 bp), a small single-copy (SSC) region (17,385 bp), and a large single-copy (LSC) region (83,538bp). The chloroplast genome encoded 133 genes, including 87 protein-coding genes, 38 tRNA genes, and 8 rRNA genes. The total GC content of the chloroplast genome was 37.55%. The phylogenetic tree showed that *X. retinervis* was closely related to *X. spekei.*

The species of genus *Xerophyta* Juss. (Velloziaceae) are known to be drought-tolerant plants (Farrant 2000). *X. retinervis* Baker 1875 is a perennial shrub up to 1.8 m tall and widely distributed through south Africa (Gibbs et al. 1987). Locally, *X. retinervis* is an extensively applied medicinal plant, with smoke from roots used to relieve asthma and smoke from the whole plant to stop nosebleeds (Van Wyk et al. 1997). Its stems are widely used to make ropes for hut and screen building, brushes, or mats in traditional home crafts (Dyer 1942).

The fresh leaves of *X. retinervis* were collected from Beijing Botanical Garden (N 39.9920, E 116.2137), Institute of Botany, Chinese Academy of Sciences, kept in silica gel, and stored at the Herbarium of Chengdu Institute of Biology (Bo Xu, xubo@cib.ac.cn) under the voucher number S1091. Total genomic DNA was extracted from dry leaves through Plant DNA Isolation Kit (Cat.No.DE-06111) and sequenced via Illumina pair-end technology. Cleaned reads were assembled using GetOrganelle v1.7.2 (Jin et al. 2020). The assembled chloroplast genome was annotated using PGA (Qu et al. 2019) and manually corrected for the start and stop codons. The annotated chloroplast genome was deposited to GenBank under the accession number MW580856.

The chloroplast genome of *X. retinervis* was 155,109 bp in length with a typical quadripartite structure, including a pair of inverted repeat regions (IRs) of 27,093 bp, a single-copy (SSC) region of 17,385 bp, and a large single-copy (LSC) region of 83,538bp. The chloroplast genome contained 133 genes, including 87 protein-coding genes, 38 tRNA genes, and 8 rRNA genes. 62% of the genes were located in the single-copy regions, and 19% were duplicated in the IR regions. The total GC content of the chloroplast genome was 37.55%.

Based on a previous study (Wanga et al. 2019), we included 21 sequences from Pandanales and two sequences from Dioscoreaceae for phylogenetic analysis. Sequences were aligned via MAFFT v7.475 (Katoh and Standley 2013). The phylogenetic tree was reconstructed using maximum-likelihood (ML) method via IQ-Tree v1.6.10 (Nguyen et al. 2015) and visualized in Figtree v1.4.4 (http://tree.bio.ed.ac.uk/software/figtree). We found that *X. retinervis* was closely related to *X. spekei* Baker 1875 in Velloziaceae clade with strong bootstrap support (Figure 1).

**Figure 1.**
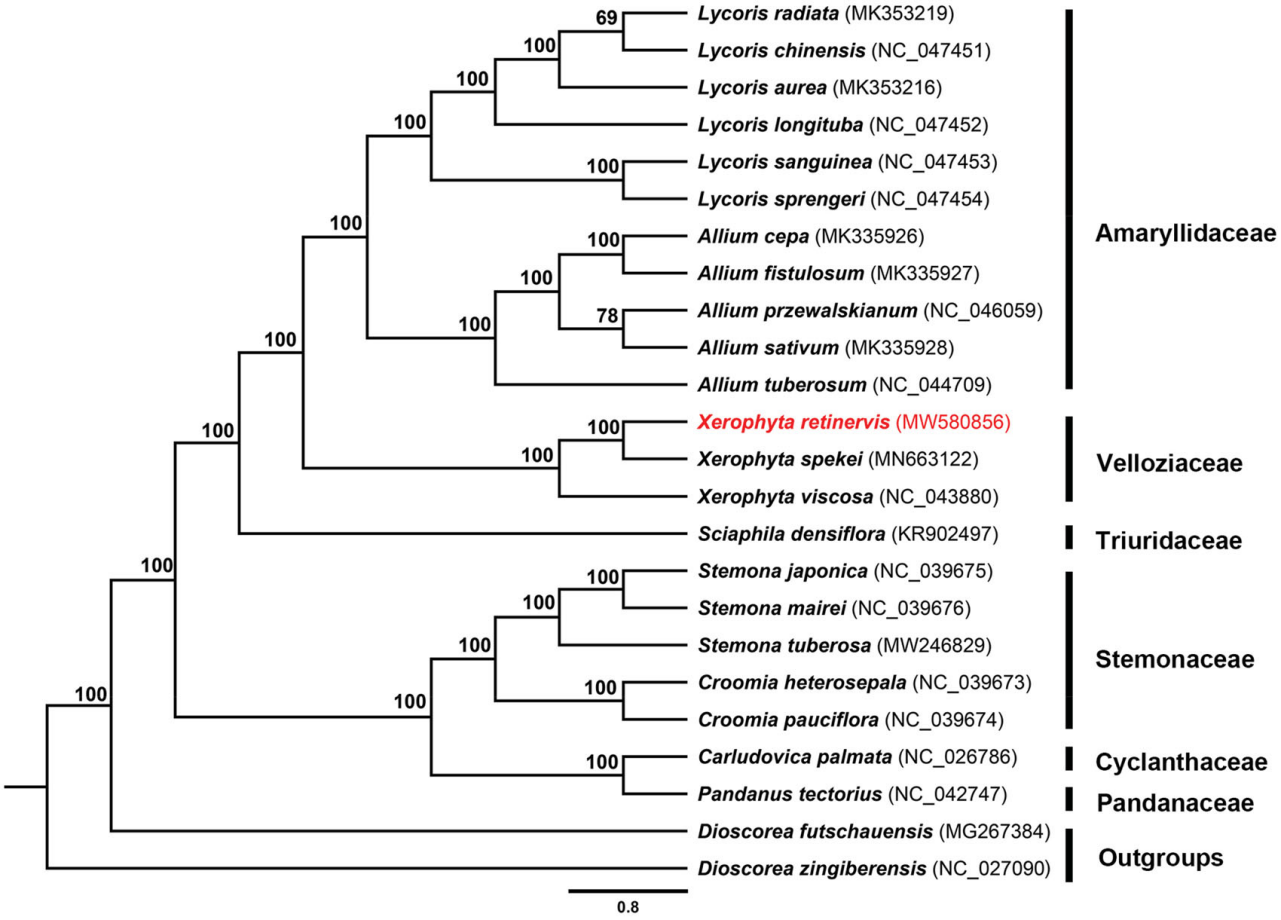
The maximum-likelihood phylogeny obtained from 24 complete chloroplast sequences.

## Data Availability

The data that support the findings of this study are openly available in GenBank number MW580856 (https://www.ncbi.nlm.nih.gov/nuccore/MW580856) and the related BioProject, raw sequencing files in SRA, and the Bio-Sample number are PRJNA810740, SRR18153545, and SAMN26278338 respectively.
